# Categorising interventions to levels of inpatient care for small and sick newborns: Findings from a global survey

**DOI:** 10.1371/journal.pone.0218748

**Published:** 2019-07-11

**Authors:** Sarah G. Moxon, Hannah Blencowe, Patricia Bailey, John Bradley, Louise Tina Day, Pavani K. Ram, Jean-Pierre Monet, Allisyn C. Moran, Willibald Zeck, Joy E. Lawn

**Affiliations:** 1 Department of Infectious Disease Epidemiology, London School of Hygiene and Tropical Medicine, London, United Kingdom; 2 Averting Maternal Death & Disability, Mailman School of Public Health, Columbia University, New York, United States of America; 3 Office of Maternal and Child Health and Nutrition, US Agency for International Development, Washington DC, United States of America; 4 Technical Division, United Nations Population Fund (UNFPA), New York, United States of America; 5 Department of Maternal, Newborn, Child and Adolescent Health, World Health Organisation, Geneva, Switzerland; 6 UNICEF Health Section, United Nations Children’s Fund (UNICEF), New York, United States of America; Centre Hospitalier Universitaire Vaudois, FRANCE

## Abstract

**Background:**

In 2017, 2.5 million newborns died, mainly from prematurity, infections, and intrapartum events. Preventing these deaths requires health systems to provide routine and emergency care at birth, and quality inpatient care for small and sick newborns. Defined levels of emergency obstetric care (EmOC) and standardised measurement of “signal functions” has improved tracking of maternal care in low- and middle-income countries (LMICs). Levels of newborn care, particularly for small and sick newborns, and associated signal functions are still not consistently defined or tracked.

**Methods:**

Between November 2016-November 2017, we conducted an online survey of professionals working in maternal and newborn health. We asked respondents to categorise 18 clinical care interventions that could act as potential signal functions for small and sick newborns to 3 levels of care they thought were appropriate for health systems in LMICs to provide: “routine care at birth”, “special care” and “intensive care”. We calculated the percentage of respondents that classified each intervention at each level of care and stratified responses to look at variation by respondent characteristics.

**Results:**

Six interventions were classified to specific levels by more than 50% of respondents as “routine care at birth,” three interventions as “special care” and one as “intensive care”. Eight interventions were borderline between these care levels. Responses were more consistent for interventions with relevant WHO clinical care guidelines while more variation in respondents’ classification was observed in complex interventions that lack standards or guidelines. Respondents with experience in lower-income settings were more likely to assign a higher level of care for more complex interventions.

**Conclusions:**

Results were consistent with known challenges of scaling up inpatient care in lower-income settings and underline the importance of comprehensive guidelines and standards for inpatient care. Further work is needed to develop a shortlist of newborn signal functions aligned with emergency obstetric care levels to track universal health coverage for mothers and their newborns.

## Introduction

Each year an estimated 2.5 million newborns die in the 28 days after birth [1]. The main causes of death are direct complications of prematurity (35%), infections (23%), and intrapartum complications leading to birth injury (24%) [2]. Most of these deaths occur in low- and middle-income countries (LMICs) [3]. Many lives could be saved—and morbidity prevented—through a health systems approach along the continuum of care [4]. Such an approach requires delivery of quality packages of care including routine and emergency care for mothers and newborns at birth, and inpatient care for small and sick newborns [4, 5].

In addition to routine essential newborn care, many low birth weight (LBW) newborns, including both preterm infants, and those born small for gestational age, require additional support to feed and to maintain their temperature [6, 7]. Preterm newborns face increased risks of respiratory problems, infections, and jaundice [8]. Even amongst those born at full term, significant numbers of newborns face complications including, systemic infections, neonatal encephalopathy, severe jaundice, and congenital disorders, with high mortality risk in the absence of quality care [3, 7]. Many of these small and sick babies will require inpatient care for them to survive and minimise chances of developing future morbidities and/or long-term disability [9–13]. Access to appropriate level and quality care remains challenging, especially for mothers and newborns experiencing complications, and notably in LMICs [4, 7, 14].

Based on evidence from higher income settings, a rational approach to organising and delivering quality services is through an integrated network of facilities providing increasing levels of care, referred to as regionalisation of care [15–17]. Managing mothers and newborns experiencing complications by more skilled staff working in specialised, better equipped facilities than in lower level facilities or those staffed solely by generalists allows for an efficient use of resources, and is an effective strategy to improve access to care for complications [14, 15, 17]. Higher levels of care build on the capabilities of lower level(s) with the additional infrastructure, equipment, supplies and health providers to manage more complex levels of care [15, 18]. For such an approach to work, synergy in institutional capabilities for mother and newborns is needed with a functional communication and referral system [15, 19, 20]. Levels of care need to be clearly defined with accompanying monitoring systems to identify issues in availability, access and quality of care for services [16, 18, 21]. Defined levels of maternal and newborn care are common in high-income settings [15, 16, 20, 22, 23], but there is a need for such a delineation for newborns in LMICs [7].

In LMICs, maternal care has been categorised by United Nations (UN) agencies at two levels referred to as basic emergency obstetric care (BEmOC) or comprehensive emergency obstetric care (CEmOC) [24]. These levels of care act as a proxy measure of the availability of the human resources, infrastructure, equipment, and supplies needed to provide specific services. This delineation allows Ministries of Health and technical partners to manage and monitor emergency obstetric care services in LMICs through “signal functions”, a core list of life-saving services that have been used to assess the provision of emergency obstetric care at either a basic or comprehensive levels [24–26]. Currently, there are seven signal functions assessed for BEmOC and two additional CEmOC signal functions; they mostly address the obstetric complications that lead to maternal death and disability, including post-partum haemorrhage, infections and hypertensive disorders [24].

Throughout this article, we will refer to the “Emergency Obstetric Care (EmOC) signal functions” in recognition of the fact that these were primarily designed from an obstetric perspective and do not represent the full spectrum of interventions required for emergency newborn care. More recently, the newborn has been more intentionally included and the term Emergency Obstetric and Newborn Care (EmONC) has emerged, a change that has been welcomed by maternal and newborn health experts, policy makers and programme implementers. We will use the term EmONC whenever we are referring to programmes, policies or indicators that were designed with a view to include both obstetric and newborn care and/or when we refer to the health facility assessments (EmONC assessments) that have been carried out with a view to looking at both maternal and newborn health services.

For small and sick newborn care in LMICs, one newborn-specific signal function, newborn resuscitation with bag and mask, was added to the core list of BEmOC signal functions nearly a decade ago [24]. However, despite the addition of a resuscitation indicator, the signal functions do not accurately represent the full package of interventions needed by the mother-baby dyad, most notably care for small and sick newborns [14, 21, 26]. This gap was highlighted by Gabrysch and colleagues in 2012, who proposed a new set of signal functions for routine and emergency maternal and newborn care following a systematic review of newborn survival literature and a consultation with 39 experts [26]. Gabrysch and colleagues proposed additional signal functions for routine and emergency care for mothers and newborns, however, this work has yet to lead to the formal definition and adoption of levels of care and accompanying newborn signal functions. Furthermore, this work did not focus intentionally on the levels of care needed for those babies born small and sick.

Since 2012, there has been a significant increase in epidemiological data for newborns [3], including better estimates of mortality, morbidity and outcomes beyond survival [8, 13, 27]. The global *Every Newborn* Action Plan, launched in 2015, called for increased focus on the programmatic and monitoring needs of newborns in order to end preventable maternal, newborn death, disability and stillbirth [21, 28, 29]. *Every Newborn* highlighted the need to improve the quality care for small and sick newborns and develop accompanying monitoring systems [3, 21]. During the past years increasingly efforts are being made by the global health community to tackle the specific health problems of small and sick newborn babies though investment in quality neonatal care. This article builds on this platform and the previous work to develop levels of care and associated signal functions [26] for small and sick newborns, in particular. The specific aim of this article is to describe the findings of an online global survey undertaken to categorise a list of newborn interventions, potential newborn signal functions, to different levels of care.

## Methods

### Study design and population

We designed an online survey to collect opinions from professionals working in maternal and newborn health, including clinicians with neonatal and obstetric experience (midwives, nurses and doctors), researchers and programme managers or governmental officials (e.g. Ministry of Health). Whilst LMIC health services for small and sick newborns was the focus, the survey was not limited to respondents based in LMICs.

### Questionnaire

We developed an online questionnaire to collect respondent characteristics (profession, current country/region of practice/employment, experience (geography, length, private/public) and type of experience (e.g. clinician, research, etc.).

We generated a list of 18 newborn services or interventions based on WHO guidelines, previous work on the subject [26] and specific work carried out as part of the *Every Newborn* process [4, 7, 30–33], including an expert focus group at an *Every Newborn* workshop where participants discussed interventions for small and sick newborns and voted on a shortlist [34]. Interventions for the shortlist were prioritised based on potential contributions to mortality reduction and LMIC health system feasibility [4, 32].

In the questionnaire, we asked respondents to assign the 18 interventions to one of 3 levels of care appropriate for health systems in LMICs to provide: “routine care at birth”, “special care”, “intensive inpatient care” as well as a classification category for services that would not be appropriate as a signal function. Routine care at birth was included based on the rationale that all newborns (including those born small and sick) will require these interventions before they are admitted to inpatient care. To avoid biasing respondents, the questionnaire generated the list of interventions/services in random order for each respondent.

The levels of inpatient care were described in the questionnaire as follows:

- Routine care at birth: This should be available at all facilities and for all babies including those that need inpatient care because they are small and sick newborns.
- Special care: this service is part of inpatient care for small and sick newborns. In many settings, this is referred to as special newborn care or level 2 care [16]. These inpatient care signal functions are interventions for small and sick newborns that should be provided in addition to routine care at birth.
- Intensive care: This service is part of inpatient care for very small and sick newborns. In most settings this will only be available at the highest level of a referral hospital. In many settings, this level is referred to as neonatal intensive care (NICU), or level 3 care [16]. These services are for very small and sick newborns in addition to all the services provided at the special care level.

We piloted the questionnaire for face validity among a group of 4 experienced public health colleagues (not part of the study team) who pre-tested and provided feedback on the question flow and wording. We then refined the wording of questionnaire based on this pilot. We translated the final version into French and Spanish, using native speakers with clinical or programmatic experience in maternal and newborn health. The final version of the questionnaire is available at http://doi.org/10.17037/DATA.00000902.

### Recruitment

The survey was accessible online for 12 months from November 2016-November 2017 in English, Spanish and French via the online platform Survey Monkey (www.surveymonkey.co.uk). Respondents could only complete the survey after giving informed consent. Respondents were given the option to exit the survey at any point. This study was granted ethical approval by the Research Ethics Committee of the London School of Hygiene &Tropical Medicine (reference number 11922).

Given that no sampling frame for this population exists, it was not possible to achieve a probability sample. Therefore, we employed a multi-faceted approach to recruit participants with diverse experience in maternal newborn health from a variety of settings, especially LMICs. We made the survey available on a wide range of professional networks, including Healthy Newborn Network https://www.healthynewbornnetwork.org/ and CHIFA http://www.hifa.org/forums/chifa-child-health-and-rights to reach both professionals working in international organisations and health professionals working on the ground. These platforms were used with the aim of recruiting a wide range of both clinicians (including nurses, midwives, doctors and allied professionals) and programme professionals with a breadth of experience. We encouraged snowball sampling by suggesting that respondents share the survey widely amongst colleagues. In addition, we promoted the survey at international conferences on newborn health: http://inkmc.net/index.php/11th-workshop-and-congress and midwifery https://www.internationalmidwives.org/events/triennial-congress/toronto-2017/.

### Statistical analyses

We calculated descriptive statistics for the respondents, including background characteristics and respondent experience. We categorised respondent experience by age group (18–34, 35–54, 55–74, 75 years or older), experience in LMICs and/or high income countries (HICs), clinical and non-clinical experience, regional base (using World Bank regions https://datahelpdesk.worldbank.org/knowledgebase/articles/906519-world-bank-country-and-lending-groups) and experience in the public and the private sector.

For each signal function:

- We calculated the percentage of respondents that classified each intervention at each level of care
- We stratified responses by respondent characteristics and looked at variation for each signal function and respondent group using chi-squared tests to identify significant differences between respondent groups and selected level of care.

## Results

### Respondent characteristics

**Fig 1:**
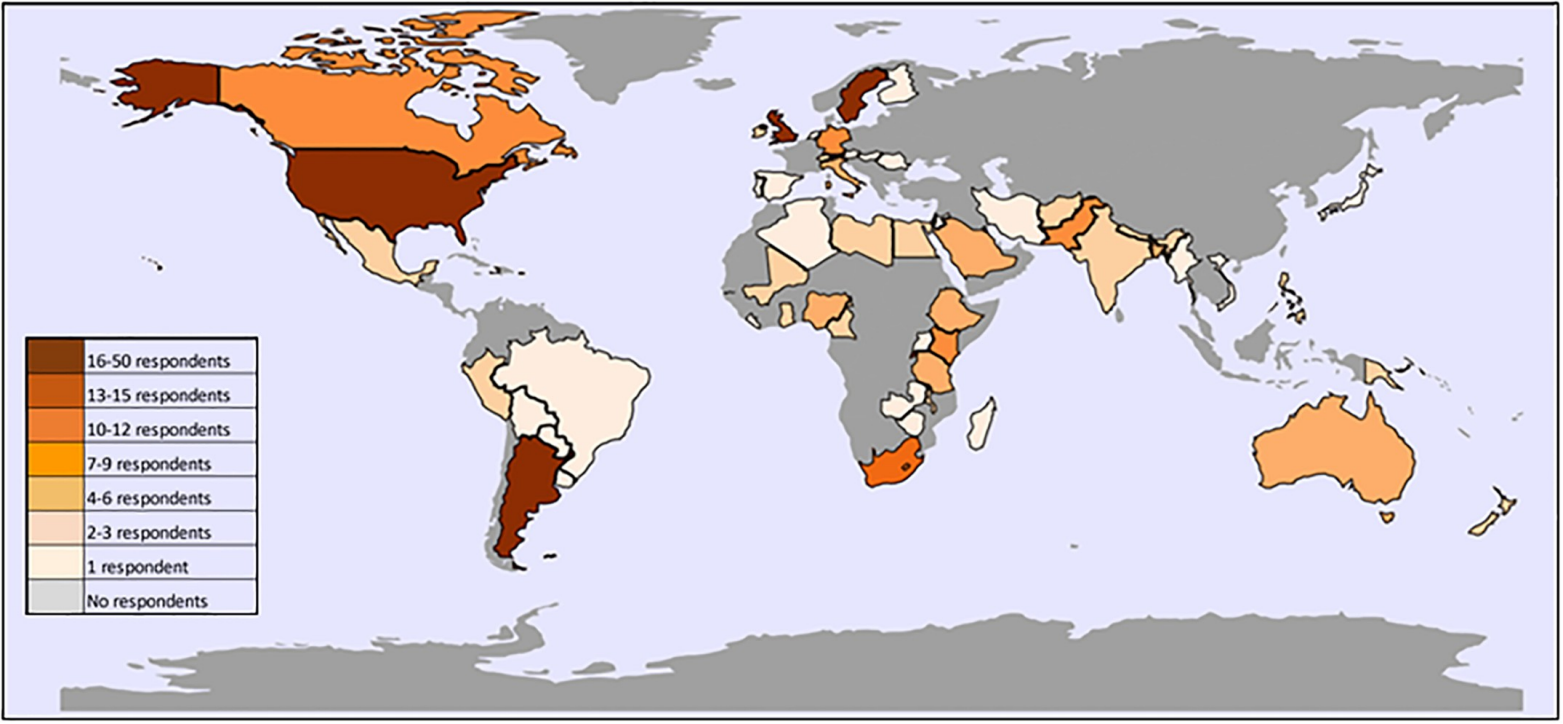
Frequency of responses to global survey on levels of inpatient care by country. Respondent experience of working in maternal and newborn health ranged from 1–49 years with a median of 19 years. The largest percentage of respondents was based in Europe and Central Asia (31%) and the smallest percentage of respondents was based in the Middle East and North Africa (5%); thereon 14% based in North America, 11% based in Latin America & Caribbean, 8% were based in South Asia, and 7% East Asia & Pacific. Over half of respondents (52%) had previous experience working in both HICs and LMICs, 13% of respondents had experience from only a high-income country and 35% only LMIC experience. The majority of respondents were trained clinicians (75%). Of these the majority were doctors (71%) followed by nurses (25%), midwives (2%) and allied health professionals (2%). Almost all clinicians had experience working in the public sector; 65% with public sector experience only, 30% with a mix of private and public-sector experience and 6% with only private sector experience.

A total of 372 individuals accessed the online survey, of which 110 (29.6%) were excluded as after registering they did not answer any questions relating to interventions and levels of care. The final sample included 262 respondents from 61 countries and 7 regions of the world (Fig 1). Data summary tables are available at http://doi.org/10.17037/DATA.00000902.

### Levels of care

**Fig 2:**
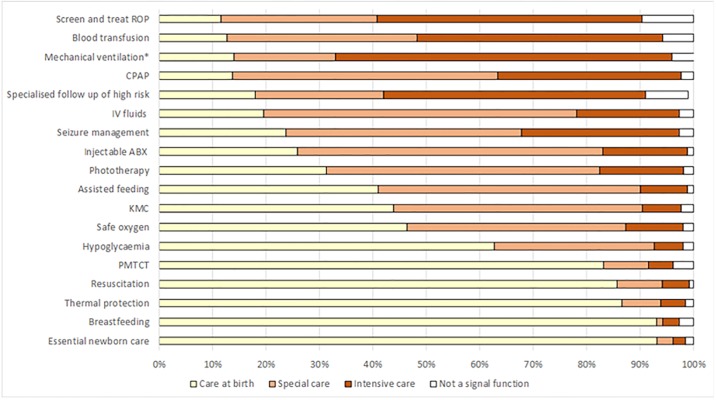
Bar graph showing list of interventions and percent of respondents for each level of care (n = 262). PMTCT = Prevention of mother to child transmission of HIV, KMC = Kangaroo mother care, ABX = antibiotics, IV = intravenous, CPAP = Continuous positive airway pressure, ROP = retinopathy of prematurity. *Only intervention classified by >50% of respondents as “intensive care”.

For the list of interventions selected as potential signal functions and the percentage of respondents that categorised these at each level of care see Fig 2.

In the following sections, we present the results by levels of care; a service was described under a specific level of care when it was selected at that level by >50% of respondents. This threshold was defined as an iterative process, based on exploration of the data.

#### Routine care at birth

Six services were selected by >50% of respondents as “routine care at birth”. Prevention of mother to child transmission of HIV (PMTCT) (83%), basic neonatal resuscitation (86%), thermal protection (87%), immediate and exclusive breastfeeding (93%), and essential newborn care (93%) were all classified by over 80% of respondents as routine care at birth. Prevention and treatment of hypoglycaemia was selected by over 60% of respondents at this level.

Other than for prevention and treatment of hypoglycaemia, we found no significant variation by respondent characteristics of the six interventions that were classified as routine care at birth. Classification of hypoglycaemia by clinicians and non-clinicians did vary significantly: 70% of clinicians classified this as care at birth compared with only 44% of non-clinicians (p<0.01). Neonatal resuscitation, the only intervention in the list that is an existing EmOC signal function, was the option with the lowest number of responses identifying it as “not a potential signal function” (1%).

#### Special care

Three services were selected by >50% of respondents as “special care”: Intravenous (IV) fluids (59%), injectable antibiotics (57%) and phototherapy (51%). Respondents with only experience in LMICs, were significantly more likely to classify IV fluids at a higher level of care compared with those with experience in a high-income setting (p<0.05). For injectable antibiotics, respondents based in high burden settings, such as South Asia, were more likely to classify this option at higher levels of care whereas Latin American respondents were more likely to classify it at a lower level (p<0.05). For phototherapy, there was some variation between non-clinicians and clinician respondents. Non-clinicians were more likely to classify phototherapy at either “special care” or “intensive care” than clinicians; 28% of non-clinicians categorised this as “intensive care” compared with only 12% of clinicians (p<0.05). A larger percentage of respondents with experience in the private sector classified phototherapy as “routine care at birth” (63%) than respondents with public (35%) or those with mixed public-private experience (27%) (p<0.05).

#### Intensive care

Mechanical ventilation was the only intervention classified by >50% of respondents as a service for “intensive care”. Respondents with experience in LMICs were more likely to classify mechanical ventilation as “intensive care” than respondents who had not worked in LMICs (66% vs. 41% respectively) (p<0.05).

#### Interventions/Services without clear categorisation (borderline)

*Borderline “routine care at birth”/ “special care” refers to interventions not meeting the >50% threshold for any level of care but categorised by close to 50% of respondents as “routine care at birth” or”special care”*. Three interventions were classified as borderline “routine care at birth”/ “special care”: safe oxygen therapy (46%/41%), KMC (44%/47%) and assisted feeding (41%/49%), respectively.

For oxygen and assisted feeding, experience of working in LMICs, regional and experience working in the public and private sector were significantly associated with variation. Those with LMIC experience or from higher burden settings were more likely to classify these interventions at higher levels of care.

For KMC, there was no significant variation between levels of care and respondent characteristics.

*Borderline* “*special care*”/ “*intensive care*” *refers to interventions not meeting the* >*50% threshold for any level of care but categorised by close to 50% of respondents as* “*special care*” *or* “*intensive care*”.

Five interventions were classified as borderline “special care”/ “intensive care”: specialised follow up of high risk (41%/49%), continuous positive airway pressure (CPAP) (50%/34%), seizure management (44%/30%), blood transfusion (36%/46%) and retinopathy of prematurity (ROP) (29%/50%).

For management of seizures, blood transfusion and CPAP, clinicians were significantly more likely to classify these interventions as special care while non-clinicians were more likely to classify them as intensive care (p<0.05).

For blood transfusion, CPAP and screening and treatment of ROP, experience in a LMIC was significantly associated with variation in the selected levels of care. Those with experience in LMICs were significantly more likely to classify these interventions as “intensive care” and while those with only experience in a high-income country more likely to classify them as “special care” (p<0.05). For example, 71% of respondents with only HIC experience classified CPAP as “special care” compared to only 33% of respondents with only LMIC experience; respondents with only LMIC experience were significantly more likely to classify it as “intensive care” (49%) (p = <0.05). For ROP, 16% of those who had only worked in LMICs responded that ROP was not a signal function compared to no respondents with only HIC experience and 7% of respondents with experience in both LMICs and HICs (p<0.05).

For specialised follow up of high risk, there was no significant variation in respondent characteristics for the between levels of care selected.

## Discussion

This article presents results from a global survey of 262 respondents from 61 countries to classify 18 newborn care interventions, into 3 levels of care. Applying the >50% threshold to 18 potential signal functions, 10 of these clearly aligned to specific levels of care: six for routine care at birth, three for special care and one for intensive care. The remaining eight signal functions did not meet the >50% threshold for a specific level of care. Previous work has encouraged the development of routine and emergency newborn signal functions [26], but levels of newborn care have not yet been well-defined for LMICs, particularly for small and sick newborns. This work contributes new insights into levels of neonatal care in LMICs as a step towards formally defined newborn care levels that could be aligned with EmOC.

### Interpretation of categorisation of levels of inpatient care for small and sick newborns from global survey

#### Consistency with existing guidelines

Out of the interventions that were clearly classified as “routine care at birth” by more than 80% of respondents, four have existing WHO guidelines (PMTCT (83%)[35], neonatal resuscitation (86%)[36], immediate and exclusive breastfeeding (93%)[37], and essential newborn care (93%) [38]). These interventions showed little variation among respondents. For more complex interventions that do not have specific WHO guidelines, level of care classification was less clear and there was greater respondent variation. This may be related to individual respondents applying existing classification systems within countries where they had worked. For example, in many settings the capacity to provide neonatal mechanical ventilation is the defining feature of an intensive care unit [39], as it requires more complex health system capacity [18]. The wording of the intervention as injectable antibiotics may have led to ambiguity with respondents by differentiating intravenous from intramuscular antibiotics. Some respondents may have perceived that intravenous infusions of antibiotics for treatment of infection may require special care capacity in contrast to intramuscular antibiotics that WHO recommends as feasible at low levels of the health system [40].

#### Low- and middle-income experience

Overall, experience in LMICs was most frequently associated with variation in response as was the case with oxygen, assisted feeding, blood transfusion, continuous positive airway pressure (CPAP) and screening and treatment for ROP. There was a clear pattern for respondents with experience in lower income settings or those based in LMICs to classify interventions more cautiously (at a higher level of care e.g. intensive care rather than special care). That respondents with LMIC experience were more comfortable assigning a higher level of care for certain interventions may reflect the respondents’ perceptions of feasibility of introducing or scaling up interventions such as CPAP [41–43]. It may also be indicative of a lack of experience delivering those interventions and/or the challenges of scaling up inpatient care in these settings. Experience in the private sector may have driven a more optimistic perception about interventions that could be provided at lower levels of care, as was the case with phototherapy, despite the increase in availability of low-cost phototherapy devices that can safely be used in LMICs [7, 44].

#### Clinical experience and knowledge of interventions

For more complex interventions, non-clinicians may not have been familiar with nomenclature, or have had less knowledge of the clinical significance or the potential feasibility of these interventions. This may explain some of the variation in responses for hypoglycaemia, treatment of seizures and phototherapy. For example, clinicians may be more likely than non-clinicians to recognise the significance of seizures in the neonatal period and the frequency of intrapartum injury in LMIC settings. The majority of respondents were clinicians who had worked in a LMIC (197/262); very few non-clinicians who had worked only in HICs (2/262) responded to the survey. However, arguably programmatic or clinical experience in LMICs was a motivating factor to respond to the survey, which related directly to LMIC health programmes and was advertised through forums relevant to these professional groups.

#### Transitional interventions

There is marked variation in the health system requirements between different levels of care. For example, facilities may be able to provide high quality routine care at birth, but lack the infrastructure, equipment and human resources to provide special care. Perceptions of the potential harm that can be caused by certain interventions if not provided in a safe, enabling environment may have influenced respondents. The perception is justified by epidemiological data showing long term consequences of poor-quality neonatal care, a pattern that has been seen in countries where there has been rapid scale up without sufficient attention to safety and monitoring systems [13, 45]. For example, countries in Asia and Latin America are seeing an epidemic of childhood blindness caused by unregulated use of oxygen in neonatal units, as well as poor screening and follow up services for survivors of neonatal care [11].

One interpretation of the results of this survey for potential signal functions that lacked clear classification may be to consider them as “transitional”. This would refer to interventions or services that bridge the nexus between two defined levels of care. This approach allows facilities that are developing inpatient care capacity at either the special care or intensive care level to go through a transitional phase whereby interventions are added in a stepwise manner before moving up to the next level of care. Facilities offering newborn care would need to offer all service category requirements at lower levels of the hierarchy before adding transitional interventions linked to higher levels [46]. For example, facilities offering routine care at birth may begin a transitional phase to building special care capacity by adding interventions such as oxygen and assisted feeding (starting with cup feeding of expressed breastmilk) in addition to or as part of stabilisation and referral. The progressive or stepwise introduction of such interventions will also be influenced by context; hospitals with larger catchment areas may need to cover a wider range of services than smaller ones. To move to the next level, all transitional interventions would need to be available and provided to a minimum standard [18].

In practice the introduction of transitional interventions would require policy and implementation discussions and further operational research, as settings differ widely [19]. Much of the existing evidence and guidance on neonatal care pertains from high income countries [15, 16, 20, 23, 46], with the majority of implementation studies from LMICs being hospital level only with few from a health systems perspective [47]. Further research is needed to document and develop quality evidence from LMICs on the organisation of neonatal care.

### Next steps: Aligning levels of inpatient newborn care with routine and emergency obstetric care measurement

**Fig 3:**
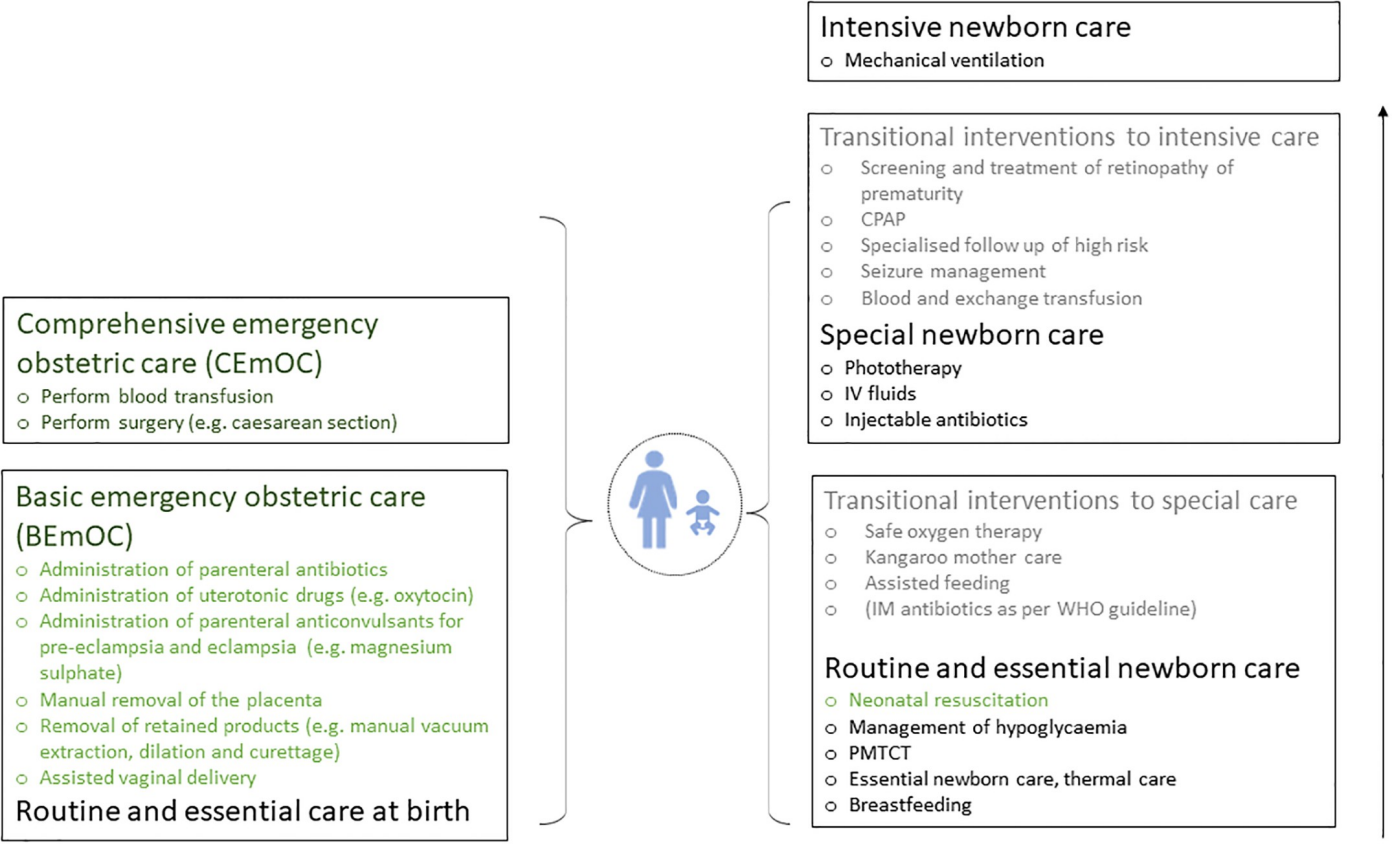
Interventions and levels of inpatient care for small and sick newborns aligned with Emergency Obstetric Care (EmOC) signal functions and levels of care. Green = existing signal function. Grey = transitional interventions. PMTCT = Prevention of mother to child transmission of HIV, IV = intravenous, IM = intramuscular, CPAP = continuous positive airway pressure.

Agreed levels of care are urgently needed for newborns, but further work is needed to align these with existing EmOC levels and determine an appropriate integrated and dynamic approach for monitoring. Fig 3 shows how the results of the survey could potentially align with the existing emergency obstetric care signal functions and levels of care. Critically, this figure places the mother and newborn together at the centre of the care.

Interventions in green are those which are existing signal functions. Starting from the bottom of the figure, routine and preventive care interventions reduce the need for emergency and inpatient care by preventing complications and there is a strong argument for their inclusion in the list of signal functions. The special care level may align with existing comprehensive signal functions as these are interventions that are only likely to be feasible to provide at a first level referral facility or regional hospital that has the capacity to have a dedicated newborn inpatient care ward and staff. A higher level, intensive care will likely only be available at a very small number of CEmOC facilities (e.g. central hospitals). Intensive care would less likely to be part of the existing EmOC framework that does not currently cover an intensive level of care for women with severe obstetric complications. These findings are also consistent with previous work that promotes the inclusion of routine and preventive care signal functions for EmoC monitoring [26]. However, one might argue that by including preventive, routine care and inpatient care measures, the framework ceases to be an “emergency framework” and becomes a framework for interventions for intrapartum and postnatal care.

Further discussion and consensus to formulate measurable newborn signal functions from this list of interventions will be needed. As currently presented, the list of interventions are potential signal functions not yet validated by being shown to link to improved outcomes, although each of these interventions does have evidence of impact [4, 7]. As part of further formative research, piloting and testing the measurement of a selection of these interventions in existing LMIC inpatient care facilities would be important. Qualitative work may be needed to look at the use of a selection of signal functions at the country and facility level in different settings. In addition, these would need to be used alongside complementary indicators that could be used for newborn care that reflect access, utilization and quality dimensions, aligned with the WHO quality of care framework [48]. The availability and density of facilities capable of providing both routine, emergency obstetric and small and sick newborn care as well as the proportion of population at a defined travel time from such facilities are useful health system tools for planning and monitoring the supply-side towards ensuring sufficient services for both maternal and newborn care. Such guidance has been lacking for small and sick newborn care, which faces major gaps in availability of and access to facilities.

This work is timely, as a revision of the EmONC monitoring handbook and associated indicators is planned. Such a revision is intended to build on lessons learned from implementation and better reflect the needs of the mother-baby dyad, including routine maternal and newborn care and inpatient care for small and sick newborns. This work contributes part of the formative work for this wider revision.

### Limitations

Since health system contexts in LMICs differ, we used an online approach to collect a wide range of opinions from different settings and professional backgrounds. However, a number of limitations of this approach must be noted. Firstly, our sample was not fully representative of all regions. Whilst the sample was geographically diverse, selection bias is a limitation and opinions of those who could not or did not access the survey due to limited internet connection, language or access issues is unknown. For example, few middle-income countries in sub-Saharan Africa were represented in the sample. The findings may also be biased by the larger frequency of respondents from Europe and the Americas, although the majority of respondents reported experience in LMIC settings even if currently based in higher-income settings. Secondly, survey fatigue may have occurred, although the list of interventions appeared in random order to avoid biasing results through respondent attrition. Survey fatigue may partially explain the number of individuals that accessed the survey but did not complete any information on newborn interventions. There may also have been a number that accessed it and realised they did not have the background knowledge to be able to answer the questions on the interventions. Several factors may have influenced the classification of inpatient care interventions, including knowledge of the intervention, perception of the importance of the intervention (e.g. its potential impact on mortality and morbidity) and perceived feasibility. This may have resulted in a conflict between perceived feasibility (can do) and perceived need (should do) and respondents may have been strongly influenced by their own personal clinical or contextual programmatic experiences. Finally, for ease of interpretation, a threshold of >50% was used to classify interventions into different levels. This was pragmatic, but entirely arbitrary threshold and the findings would be slightly different if other thresholds were applied.

This work was focused on inpatient care for small and sick newborns that occurs in the postnatal period. It does not discuss community interventions or interventions that benefit newborns but are delivered in the antenatal period. The use of antenatal corticosteroids for mothers with threatened preterm labour [49] and antibiotics for preterm premature rupture of membranes (P-PROM) [50] are two interventions for small and sick newborns that have an evidence base, but that do not naturally fit into the inpatient newborn care package due to the timing in the peripartum period.

## Conclusions

This article has shown how practitioners categorised 18 newborn interventions that could act as potential signal functions to different levels of care, including routine care at birth and inpatient care for small and sick newborns. Findings were consistent with existing clinical guidelines and previous work on the subject, but also provided new insights on how newborn care programmes, including more complex interventions for small and sick newborns, could be organised and monitored. Future research should focus on refining the list to a small selection of measurable signal functions and testing of these potential signal functions in existing inpatient care units. Further work is needed to align these newborn signal functions to the existing obstetric care levels to create a dynamic and integrated framework for maternal and newborn care. Working towards universal health coverage, future adaptations, including improvements to indicators of service availability, access and quality, should reflect the needs of health programmes for both mothers and their newborns.
